# Dual Use of a Patient Portal and Clinical Video Telehealth by Veterans with Mental Health Diagnoses: Retrospective, Cross-Sectional Analysis

**DOI:** 10.2196/11350

**Published:** 2018-11-07

**Authors:** Erica A Abel, Stephanie L Shimada, Karen Wang, Christine Ramsey, Melissa Skanderson, Joseph Erdos, Linda Godleski, Thomas K Houston, Cynthia A Brandt

**Affiliations:** 1 Pain Research, Informatics, Multimorbidities and Education Center VA Connecticut Healthcare System West Haven, CT United States; 2 Yale Center for Medical Informatics Yale School of Medicine New Haven, CT United States; 3 Department of Psychiatry Yale School of Medicine New Haven, CT United States; 4 Center for Healthcare Organization and Implementation Research Edith Nourse Rogers Memorial Veterans Hospital Bedford, MA United States; 5 Department of Health Law, Policy, and Management Boston University School of Public Health Boston, MA United States; 6 Division of Health Informatics and Implementation Science, Department of Quantitative Health Sciences University of Massachusetts Medical School Worcester, MA United States; 7 Department of Internal Medicine Yale School of Medicine New Haven, CT United States; 8 National Telemental Health Center VA Connecticut Healthcare System West Haven, CT United States; 9 Department of Emergency Medicine Yale School of Medicine New Haven, CT United States

**Keywords:** mental health, patient portals, telemedicine, telehealth, eHealth, United States Department of Veterans Affairs

## Abstract

**Background:**

Access to mental health care is challenging. The Veterans Health Administration (VHA) has been addressing these challenges through technological innovations including the implementation of Clinical Video Telehealth, two-way interactive and synchronous videoconferencing between a provider and a patient, and an electronic patient portal and personal health record, My HealtheVet.

**Objective:**

This study aimed to describe early adoption and use of My HealtheVet and Clinical Video Telehealth among VHA users with mental health diagnoses.

**Methods:**

We conducted a retrospective, cross-sectional analysis of early My HealtheVet adoption and Clinical Video Telehealth engagement among veterans with one or more mental health diagnoses who were VHA users from 2007 to 2012. We categorized veterans into four electronic health (eHealth) technology use groups: My HealtheVet only, Clinical Video Telehealth only, dual users who used both, and nonusers of either. We examined demographic characteristics and mental health diagnoses by group. We explored My HealtheVet feature use among My HealtheVet adopters. We then explored predictors of My HealtheVet adoption, Clinical Video Telehealth engagement, and dual use using multivariate logistic regression.

**Results:**

Among 2.17 million veterans with one or more mental health diagnoses, 1.51% (32,723/2,171,325) were dual users, and 71.72% (1,557,218/2,171,325) were nonusers of both My HealtheVet and Clinical Video Telehealth. African American and Latino patients were significantly less likely to engage in Clinical Video Telehealth or use My HealtheVet compared with white patients. Low-income patients who met the criteria for free care were significantly less likely to be My HealtheVet or dual users than those who did not. The odds of Clinical Video Telehealth engagement and dual use decreased with increasing age. Women were more likely than men to be My HealtheVet or dual users but less likely than men to be Clinical Video Telehealth users. Patients with schizophrenia or schizoaffective disorder were significantly less likely to be My HealtheVet or dual users than those with other mental health diagnoses (odds ratio, OR 0.50, CI 0.47-0.53 and OR 0.75, CI 0.69-0.80, respectively). Dual users were younger (53.08 years, SD 13.7, vs 60.11 years, SD 15.83), more likely to be white, and less likely to be low-income than the overall cohort. Although rural patients had 17% lower odds of My HealtheVet adoption compared with urban patients (OR 0.83, 95% CI 0.80-0.87), they were substantially more likely than their urban counterparts to engage in Clinical Video Telehealth and dual use (OR 2.45, 95% CI 1.95-3.09 for Clinical Video Telehealth and OR 2.11, 95% CI 1.81-2.47 for dual use).

**Conclusions:**

During this study (2007-2012), use of these technologies was low, leaving much potential for growth. There were sociodemographic disparities in access to My HealtheVet and Clinical Video Telehealth and in dual use of these technologies. There was also variation based on types of mental health diagnosis. More research is needed to ensure that these and other patient-facing eHealth technologies are accessible and effectively used by all vulnerable patients.

## Introduction

### Background

Veteran access to mental health care within the US Department of Veterans Affairs Health Administration (VHA), especially for rural veterans, has been a challenge [1]. About 2.9 million, 56% of all rural veterans, are enrolled in VHA care [2]. As compared with urban veterans, rural veterans with mental health diagnoses are sicker and face challenges accessing health care, including stigma, increased distance needed to travel to care, lack of access to transportation, and lack of specialists or providers in rural areas [3,4]. Although the VHA offers mental health services nationally through a combination of regional medical centers and community-based outpatient clinics, some locations may not have the specialty services or the staff to meet the demand for needed services [5].

To address access barriers and to enhance patient care, the VHA has been transforming the provision of clinical care, in part, through technological innovation. The VHA has been a leader nationally through its deployment of several health information technologies [6], including an integrated electronic medical record, Home Telehealth (eg, in-home, messaging, and peripheral devices such as blood pressure and heart rate monitors), mobile health apps, Clinical Video Telehealth (CVT), and an integrated Web-based personal health record and patient portal, My HealtheVet (MHV). These patient-facing technologies are consistent with recommendations by the Institute of Medicine to support continuous healing relationships through use of the internet and technologies that provide patients with access to care outside of face-to-face visits, and access to their medical information, when and where they need it most [7]. They also offer unique ways for patients and providers to communicate in addition to or in lieu of traditional face-to-face encounters [8]. The concept of *complementary use* of health care technology —the use of features and functionality of two different technologies in conjunction with one another—is a way of enhancing access to care and supporting patient-centered care by supporting communication, information sharing, and increasing patient involvement in their care [8]. The integration of technologies in a complementary way has the potential to increase veterans’ access to care and improve the quality of delivered care. Dual use of technologies—veteran adoption and use of more than one technology at any point—may be a precursor to *complementary use*. For example, veterans who have adopted the MHV portal and have engaged in CVT might be willing or inclined to use both tools in a complementary way.

### History of My HealtheVet and Clinical Video Telehealth

MHV and CVT are two established virtual care technologies in the VHA. Over a decade ago, the VHA launched its Web-based personal health record and patient portal, My HealtheVet (MHV), to complement traditional health care services, to improve comanaged care, and to promote active engagement of patients and their families in the patient’s health care [9]. The MHV portal allows users to create and maintain a comprehensive personal health record by using a range of MHV features, including secure messaging, Web-based prescription refills, access to information in their VHA health record (eg, laboratory results, clinical progress notes, discharge summaries, and medication lists), and tracking of personal and self-reported health information using a variety of tools (eg, food, activity and allergy journals, family health history, and other data). Access to features depends on the type of MHV enrollment and account type. MHV registration creates a basic account that provides access to the MHV self-report features (ie, self-entered information or journals). An advanced account is limited to veterans and/or VHA patients and gives these users the ability to refill prescriptions and to view some of their information in their health records. A premium account, also only for veterans or VHA patients, is the highest level of MHV access and requires users to verify their identity either in-person or on the Web, a process known as authentication. In addition to using MHV to view many parts of their VHA health record and Department of Defense Military Service Information, premium users can send secure messages to communicate with their health care providers and health care teams [10]. As of July 2018, of the 4.45 million who registered (since November 2004), about 3.9 million indicated they were veterans. Of the 4.45 million registrants, about 2.74 million have authenticated since January 1, 2007 [11].

The VHA has also expanded access to health care through a range of telehealth services, including CVT which is two-way interactive and synchronous videoconferencing between a provider and a patient at a distance in settings such as a VHA medical center and a community clinic or home. CVT is available across a range of medical and mental health specialties. Unlike MHV, a Web app, access to CVT is less ubiquitous for two primary reasons: (1) clinicians refer patients and place consults to CVT programs (patients may not be able to self-refer) and (2) CVT programs that do exist are not available to every veteran, everywhere. Some programs are regional—they emanate from a regional medical center or hub and provide remote specialty services to clinics that are closer to a veteran’s home, thus reducing travel time and time away from work or family. A few programs provide services across state lines, although to specific sites (the provider and veteran must be located in a federal facility), and some programs provide CVT into the home.

Devices used in CVT within VHA are often attached to computers (eg, video cameras and microphones) or are stand-alone (eg, videoconferencing equipment). For visits conducted at a VHA medical center or community-based clinic, a VHA employee at the patient site (known as a telehealth clinical technician [TCT]) seats the patient in the CVT-equipped space and coordinates initiation of the CVT call with the provider. The TCT then leaves the room and is available as needed to provide technical or administrative support throughout the clinical encounter. If the patient is at home, the process is different as a TCT is not involved. The provider and the patient connect with each other using preplanned processes. Whether CVT is home- or clinic-based, the providers have well-defined, documented, and tested emergency procedures available.

There is evidence that veterans with mental health conditions are interested in, and have used, both MHV and CVT. Among veterans who reported enrollment and mental health use data in the 2010 National Survey of Veterans, 25% of those who indicated they used VHA mental health services endorsed that they used MHV to obtain information about their personal VHA health care [12]. In a study of veterans receiving care in VHA, those with trauma-related conditions and common mental health conditions (eg, depression, bipolar disorder, or posttraumatic stress disorder [PTSD]) were among the highest early adopters of MHV. The adoption of MHV among veterans with depression, anxiety, and PTSD was high compared with patients with other diagnoses [13].

In addition, VHA CVT services for individuals with mental health conditions (known also as telemental health) have grown tremendously. In October 2002, VHA began coding telehealth activity distinctly to enable its measurement. Between October 2002 and August 2018, there were more than 3.75 million CVT telemental health patient encounters. In 2017, VHA delivered more than 470,000 CVT telemental health patient encounters to over 150,000 unique patients [14]. During this study period (2007-2012), there were over 320,000 unique CVT users.

Recent research has explored whether veterans with mental health diagnoses are willing to use different electronic health (eHealth) technologies, including CVT. A survey of veterans in Hawaii (VHA and non-VHA users) found that 32% to 57% of those surveyed were receptive to using different technologies in their mental health care (ie, telephone calls, CVT into the home or clinic, Web-based computer-based interventions, personalized messages to computer, short message service (SMS) text messages to cell phone, or social networking with a peer group). Veterans’ willingness varied and depended on the technology and their PTSD screen status [15]. Veterans with probable PTSD were significantly less likely than those with no PTSD to report willingness for CVT in clinic (20.4% vs 45.6%) or CVT in the home (25.5% vs 52.7%). The survey did not include questions about use of specific MHV features.

Research also has examined veterans’ preferences on their use of technologies in managing their health care [16]. Whealin et al conducted a survey of a random sample of veterans who received VHA care and who had registered to use MHV [16]. Among those with a PTSD diagnosis and at least one chronic medical condition, 44.6% used health-related technology 1 to 3 times per month and 21.4% used it less than once per month. Most common uses of the technologies included searching for health information (78.9%), communicating with providers (71.1%), and tracking medications (64.9%). Respondents reported they were most experienced and comfortable with using computers, the Web, email (99%-100%) and had less experience and comfort with other modes, including social media (73.0%), mobile apps (79.6%), and Clinical Video Telehealth (67.3%).

Despite these early studies, there has been little work examining the extent to which veterans might be using and benefitting from the use of multiple eHealth technologies. This information is increasingly important as the VHA strives to improve access for all veterans, especially those with mental health conditions. Information on whether and how veterans use these two technologies provides information that could be leveraged to identify additional opportunities for mental health–related treatments and interventions, improve access to and patient engagement in mental health treatment, and improve the overall quality of mental health care. To our knowledge, research has not yet examined veterans’ use of multiple eHealth technologies on a large scale using administrative data. This study examines (1) the early adoption and feature use of MHV, engagement in CVT, and the dual use of these two technologies and (2) the sociodemographic and mental health characteristic associated with the use of these technologies. The use of two technologies may lay the groundwork for and lead to the complementary use of technologies.

## Methods

### Study Design

We conducted a cross-sectional analysis of veterans’ use of two VHA technologies: (1) the MHV personal health record and patient portal and (2) Clinical Video Telehealth.

### Study Population

The sample for this study (N=2,171,325) is drawn from a retrospective cohort study evaluating technology adoption in VHA users [13]. This sample includes all veterans aged between 18 and 100 years, who received inpatient or outpatient care at VHA during our study period of October 1, 2007, through March 31, 2012, and had one or more common or high-priority mental health diagnoses by the time of MHV registration or first CVT visit (see variable: mental health conditions). We chose to study this early period (ending March 2012), which coincided with the initial rollout of secure messaging to providers and patients. The study period overlaps with both the MHV pilot period (October 2007 and October 2009) and the 2010 to 2012 early national rollout of MHV and secure messaging. The Human Research Protection Program at the Veterans Affairs (VA) Connecticut Healthcare System and the Yale School of Medicine and Institutional Review Board at the Edith Nourse Rogers Memorial Veterans Hospital in Bedford, Massachusetts, approved this study.

### Data Sources

Administrative, clinical, CVT, and MHV data for October 1, 2007, through March 31, 2012, the study period, were pulled from the VHA Corporate Data Warehouse. Variables extracted include patient demographics, medical and mental health diagnoses, MHV enrollment and authentication statuses, use of certain MHV features (ie, secure messaging and prescription refill), and CVT engagement.

### Variables

#### Dependent Variables: Electronic Health Technology Use Groups—Dual Use, My HealtheVet Adoption, Clinical Video Telehealth Engagement

We created indicators for MHV adoption, CVT engagement, and dual use as dependent variables in our multivariable logistic regression models. To establish MHV adoption, we used MHV data on registration, authentication, and feature use. The available data included flags for adoption such as MHV *registration* (ie, the process of creating a personal profile, log-in, and access account to gain access to MHV), *authentication* (ie, the verification of identity before granting access to personal health information), and MHV *feature use,* including secure messaging use (ie, ever sent or ever read a secure message) and Web-based prescription refill (ie, ever refilled prescriptions on the Web). To establish CVT engagement, we identified whether the patient had ever had a CVT visit during the study period. A patient was determined to be a dual user if he or she had adopted MHV and engaged in CVT during the study period; the MHV adoption and CVT engagement did not have to be concurrent.

For our other analyses, we grouped veterans into four mutually exclusive eHealth technology use groups based on their MHV adoption and their CVT engagement (*CVT-MHV groups*): (1) dual users—veterans who had adopted MHV and had engaged in CVT at any point during the study period; (2) MHV only—veterans who had adopted MHV and did not have a CVT visit; (3) CVT only—veterans who had a CVT visit for mental health during the study period but had no MHV adoption; and (4) neither—veterans who had neither adopted MHV nor engaged in CVT during the study period.

#### Independent Variables: Mental Health Conditions

We focused on common or high priority mental health conditions in the veteran population, including bipolar disorder, major depression, other depression (ie, depressive disorders not meeting criteria for major depressive disorder such as adjustment disorder, depression not otherwise specified), anxiety, PTSD, schizophrenia or schizoaffective disorder, and other psychotic disorders. Veterans in the full study cohort may have multiple mental health diagnoses. We used previously validated diagnostic code groupings [17] and ascertained from the administrative data if veterans had one or more of the mental health diagnoses documented during the study period. We counted mental health conditions coded at least once for an inpatient stay or at least twice for an outpatient visit during the study period. Prior research has demonstrated that this approach improves the accuracy of the identification of disorders in administrative data [18,19] because outpatient codes are assigned by health care providers and may be less accurate than inpatient codes, which are assigned by professional coders in the VHA. Diagnoses were classified according to *International Classification of Diseases, Ninth Revision, Clinical Modification.*

#### Covariates: Demographic Characteristics

Demographic variables included gender, age, race or ethnicity, rural residence, and economic need. Rural residence was determined based on zip code of residence using VHA Office of Rural Health definitions based on the Rural-Urban Commuting Areas system (ie, urban, rural, and highly rural) [20]. As a proxy for socioeconomic status to capture economic need, we created a flag to indicate patients who qualified for free VHA health care based on a financial assessment.

### Analyses

We used descriptive statistics to examine sociodemographic and mental health characteristics for the full cohort and within each of the four eHealth technology groups. We compared MHV feature use between the MHV only and dual users groups using chi-square statistics. In addition, among MHV adopters (MHV only and dual users), we examined the proportion of veterans in the full cohort and within each of the diagnostic code groupings who used any features of MHV (any MHV) and who used two specific features of MHV: prescription refills and secure messaging.

We used separate multivariable logistic regression models to examine predictors of MHV adoption versus nonadoption, CVT engagement versus nonengagement, and dual use versus nondual use. We examined associations between mental health diagnoses and eHealth technology use, adjusting for patient sociodemographic characteristics previously shown to be significantly associated with MHV use [13], and accounted for clustering of veterans within VHA health care regions (ie, Veterans Integrated Service Networks [VISNs]) by including VISN as a random effect in the models. SAS 9.4 was used to run all analyses (SAS institute, Cary, NC).

## Results

### Patient Demographics and Electronic Health Technology Adoption

From a cohort of over 6 million active users of the VHA, 2,171,325 patients aged between 18 and 100 years had one or more of the mental health conditions. As shown in Table 1, the majority were male, white and resided in urban areas. Mean age was 60.11 (SD 15.83), and over a quarter qualified for free care in VHA based on economic need. Among these 2.17 million veterans, 1.51% (32,723/2,171,325) were dual users of MHV and CVT. Most patients (1.56 million) had neither adopted or used MHV features or engaged in CVT (71.72%, 1,557,218/2,171,325); 23.00% (499,445/2,171,325) of the patients were MHV only users and 3.77% (81,939/2,171,325) had engaged in CVT only. Compared with the overall cohort, dual users included a larger proportion of women veterans (8.49%, 184,331/2,171,173 vs 12.8%, 4212/32,721), white patients (77.70%, 1,521,828/1,958,625), and patients from rural areas (25.73%, 545,755/2,121,406 vs 44.27%, 14,256/32,199). Compared to the overall cohort, dual users had a lower mean age 53.08 (SD 13.74) years versus 60.11 (SD 15.83) years and a lower percentage of individuals with high economic need (26.15%, 567,728/2,170,948 vs 17.56%, 5746/32,723).

Over a third of the entire cohort had a diagnosis of PTSD (36.47%, 791,839/2,171,325), 31.47% (683,268/2,171,325) had a diagnosis of anxiety disorder, 23.95% (520,088/2,171,325) had a diagnosis of major depression, 17.61% (382,438/2,171,325) had other psychotic disorder diagnoses, and 12.68% (275,331/2,171,325) had a diagnosis of bipolar disorder (Table 2). Nearly two-thirds of individuals in the entire cohort (62.41%, 1,355,039/2,171,325) had other depression diagnoses. CVT engagement and MHV use varied by mental health diagnosis. Compared with veterans with other psychotic or schizophrenia or schizoaffective disorders, those with a diagnosis of depression, PTSD, anxiety, or bipolar disorder were higher users of both MHV and CVT.

The unadjusted comparisons of MHV feature use between MHV only and dual users show some differences (Table 3). Significantly more dual users authenticated or ever filled a prescription on the Web than MHV only users. There were no differences between dual and MHV users in secure messaging use.

**Figure 1:**
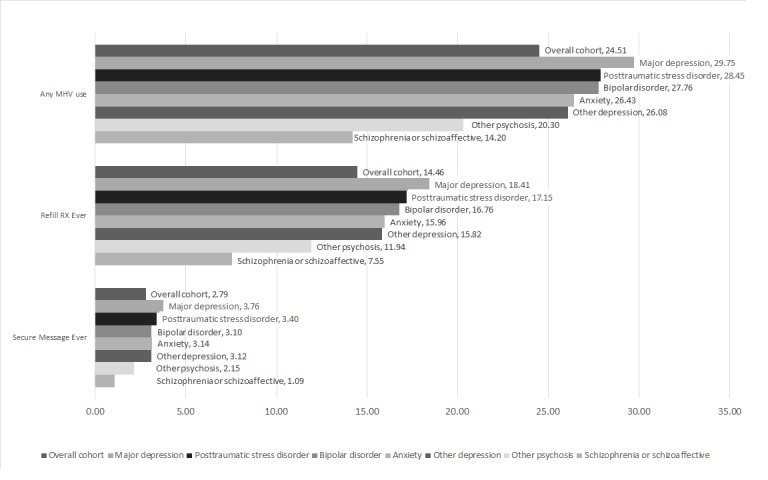
My HealtheVet use by mental health diagnosis. MHV: My HealtheVet; RX: Prescription.

As shown in Figure 1, there were variations in use of MHV and its primary features across mental health diagnoses. The percentages shown in the figure reflect the overall proportion of patients with that diagnosis who engage in each type of use. Individuals with major depression, PTSD, or bipolar diagnoses had overall higher levels of any MHV feature use and used the prescription refill feature more often than individuals with other diagnoses. Individuals diagnosed with schizophrenia or schizoaffective disorders and other psychotic disorders had consistently lower levels of any MHV feature use, prescription refill, and secure message use.

**Table 1:** Demographics by electronic health technology use groups.

Demographics^a^		Full cohort (N=2,171,325)	Dual users (n=32,723)	MHV^b^ only (n=499,445)	CVT^c^ only (n=81,939)	Neither (n=1,557,218)
Gender (male), n (%)		1,986,842 (91.51)	28,509 (87.13)	435,459 (87.19)	75,733 (92.44)	1,447,141 (92.94)
**Residence, n (%)**						
	Urban	1,575,651 (74.27)	27,954 (55.73)	382,066 (78.13)	40,298 (49.97)	1,135,344 (74.71)
	Rural	545,755 (25.73)	14,256 (44.27)	106,920 (21.87)	40,342 (50.03)	384,237 (25.29)
**Race or ethnicity, n (%)**						
	White	1,521,828 (77.70)	26,075 (86.20)	373,057 (81.93)	61,208 (82.13)	1,061,488 (75.90)
	African American	364,107 (18.59)	3116 (10.30)	65,704 (14.43)	10,206 (13.69)	285,081 (20.38)
	Latino	12,813 (0.65)	114 (0.38)	2048 (0.45)	445 (0.60)	10,206 (0.73)
	Other^d^	59,877 (3.06)	943 (3.12)	14,533 (3.19)	2669 (3.58)	41,732 (2.98)
High economic need, n (%)		567,728 (26.15)	5746 (17.56)	98,779 (19.78)	20,545 (25.07)	442,658 (28.43)
**Age (years)**, **n (%)**						
	<40	277,140 (12.76)	6712 (20.51)	81,624 (16.34)	12,632 (15.42)	176,172 (11.31)
	40 to 59	653,918 (30.12)	12,770 (39.02)	179,169 (35.87)	27,136 (33.12)	434,843 (27.92)
	60 to 79	985,240 (45.38)	12,696 (38.80)	213,008 (42.65)	37,585 (45.87)	721,951 (46.36)
	>80	255,027 (11.75)	545 (1.67)	25,644 (5.13)	4586 (5.60)	224,252 (14.40)
Age (years), mean (SD)		60.11(15.83)	53.08 (13.74)	56.18 (14.68)	57.02 (14.70)	61.67 (16.00)

^a^Numbers may not sum because of missing data, and percentages may not sum to 100% because of rounding. The listed column percentages exclude missing data.

^b^MHV: My HealtheVet.

^c^CVT: Clinical Video Telehealth.

^d^Other category includes American Indian, Asian, and Native Hawaiian.

**Table 2:** Mental health conditions by electronic health technology use groups.

Mental health conditions	Full cohort, n^a^ (%)	Dual users, n (%)	MHV^b^ only, n (%)	CVT^c^ only, n (%)	Neither, n (%)
Other depression	1,355,039 (62.41)	23, 679 (1.75)	329,706 (24.33)	56,414 (4.16)	945,240 (69.76)
Posttraumatic stress disorder	791,839 (36.47)	18,611 (2.35)	206,644 (26.10)	41,036 (5.18)	525,548 (66.37)
Anxiety	683,268 (31.47)	13,209 (1.93)	167,405 (24.50)	30,799 (4.51)	471,855 (69.06)
Major depression	520,088 (23.95)	12,868 (2.47)	141,864 (27.28)	26,937 (5.18)	338,419 (65.07)
Bipolar disorder	275,331 (12.68)	6666 (2.42)	69,764 (25.34)	15,436 (5.61)	183,465 (66.63)
Other psychotic disorders	382,438 (17.61)	5073 (1.33)	72,563 (18.97)	14,690 (3.84)	290,112 (75.86)
Schizophrenia or schizoaffective	124,879 (5.75)	1229 (0.98)	16,506 (13.22)	6261 (5.01)	100,833 (80.78)

^a^Veterans may have multiple mental health diagnoses; therefore, percentages do not sum to 100%.

^b^MHV: My HealtheVet.

^c^CVT: Clinical Video Telehealth.

**Table 3:** My HealtheVet adoption and feature use with and without dual use (N=2,171,325).

My HealtheVet feature use	Dual users (n=32,723), n (%)^a^	MHV^b^ only (n=449,445), n (%)^a^	*P*^c^ value
Authenticated	23,573 (72.04)	347,610 (69.60)	<.001
Ever filled a prescription on the Web	20,589 (63.88)	293,409 (59.59)	<.001
Ever used secure messaging^d^	3561 (10.88)	57,126 (11.44)	.002

^a^The total number of Veterans in each group and the percent of the overall study population.

^b^MHV: My HealtheVet.

^c^*P* value for chi-square.

^d^Only authenticated users who opt-in can secure message; however, the denominator for the percentage calculation is based on the total number of dual users or MHV only users to show the overall penetration of secure messaging activity among the entire population in each column.

**Table 4:** Adjusted odds ratios of My HealtheVet, Clinical Video Telehealth, and dual use based on demographic characteristics (N=1,911,085).

Demographic characteristics		Model^a^ predicting MHV^b^ adoption, OR^c^ (95% CI)	Model^a^ predicting CVT^d^ engagement, OR (95% CI)	Model^a^ predicting dual use of both MHV and CVT, OR (95% CI)
**Age in years**				
	<40	Reference	Reference	Reference
	40 to 59	1.04 (1.01-1.07)	0.91 (0.87-0.95)	0.91 (0.88-0.94)
	60 to 79	0.70 (0.65-0.75)	0.71 (0.65-0.77)	0.56 (0.51-0.61)
	>80	0.27 (0.24-0.30)	0.40 (0.31-0.51)	0.17 (0.13-0.21)
**Gender**				
	Female	1.57 (1.51-1.62)	0.92 (0.89-0.96)	1.16 (1.11-1.20)
	Male	Reference	Reference	Reference
**Race or ethnicity**				
	African American	0.51 (0.48-0.54)	0.72 (0.62-0.85)	0.51 (0.46-0.57)
	Latino	0.53 (0.46-.062)	0.88 (0.79-0.98)	0.58 (0.41-0.82)
	Other	0.83 (0.78-0.89)	0.98 (0.90-1.06)	0.82 (0.76-0.89)
	White	Reference	Reference	Reference
**Residence**				
	Rural	0.83 (0.80-0.87)	2.45 (1.95-3.09)	2.11 (1.81-2.47)
	Urban	Reference	Reference	Reference
**Income**				
	High economic need	0.64 (0.63-0.66)	0.99 (0.96-1.02)	0.75 (0.71-0.79)
	Other	Reference	Reference	Reference
**Bipolar disorder**				
	Yes	1.09 (1.07-1.12)	1.43 (1.35-1.51)	1.45 (1.37-1.53)
	No	Reference	Reference	Reference
**Major depression**				
	Yes	1.25 (1.22-1.28)	1.38 (1.26-1.52)	1.56 (1.45-1.68)
	No	Reference	Reference	Reference
**Posttraumatic stress disorder**				
	Yes	1.19 (1.16-1.22)	1.74 (1.58-1.91)	1.86 (1.77-1.96)
	No	Reference	Reference	Reference
**Schizophrenia or schizoaffective disorder**				
	Yes	0.50 (0.47-0.53)	1.25 (1.17-1.33)	0.75 (0.69-0.80)
	No	Reference	Reference	Reference
**Other psychosis**				
	Yes	1.04 (1.01-1.07)	1.14 (1.10-1.19)	1.13 (1.08-1.19)
	No	Reference	Reference	Reference
**Other depression**				
	Yes	1.20 (1.18-1.22)	1.32 (1.24-1.40)	1.42 (1.34-1.51)
	No	Reference	Reference	Reference
**Anxiety**				
	Yes	1.09 (1.07-1.11)	1.25 (1.17-1.33)	1.27 (1.20-1.35)
	No	Reference	Reference	Reference

^a^Models accounted for clustering of veterans within VHA health care regions (known as Veterans Integrated Service Networks [VISN]) by including VISN as a random effect to adjust for VISN-level differences.

^b^MHV: My HealtheVet.

^c^OR: odds ratio.

^d^CVT: Clinical Video Telehealth.

### Models Predicting Adoption of My HealtheVet, Clinical Video Telehealth, and Dual Use

#### Sociodemographic Characteristics Associated With My HealtheVet, Clinical Video Telehealth, and Dual Use

Our logistic regression models showed that holding all other demographic characteristics and diagnoses constant, age is a strong predictor of any type of technology use (Table 4). Veterans with mental health diagnoses who were over the age of 60 years had significantly lower odds of MHV adoption, CVT engagement, or dual use than those under the age of 40 years. For example, the odds of a veteran being a dual user were 44% lower for those aged 60 to 79 years (OR 0.56, 95% CI 0.51-0.61) and 83% lower for those aged over 80 years (OR 0.17, 95% CI 0.13-0.21) compared with those aged under 40 years. The odds of veterans aged 40 to 59 years adopting MHV were 4% higher than the odds for veterans under 40 years (OR 1.04, 95% CI 1.01-1.07); however, those aged 40 to 59 years were less likely to engage in CVT than those aged under 40 years (OR 0.91, 95% CI 0.87-0.95).

There were also differences in technology use for other demographic characteristics. Women, as compared with men, had higher odds of both MHV adoption and dual use (OR 1.57, 95% CI 1.51-1.62 for MHV and OR 1.16, 95% CI 1.11-1.20 for dual use) but slightly lower odds of CVT engagement (OR 0.92, 95% CI 0.89-0.96). African American and Latino veterans had significantly lower odds of MHV adoption, CVT engagement, or dual use as compared with white veterans using the same technologies. The odds of an African American veteran adopting MHV, engaging in CVT, or being a dual user were 49%, 28%, and 49% lower, respectively, whereas those of Latino veteran were 47%, 12%, and 42% lower, respectively. Although being low income did not predict CVT engagement, low-income veterans eligible for free VHA care based on income had odds of MHV adoption that were 36% lower (OR 0.64, 95% CI 0.63-0.66) and odds of dual use that were 25% lower (OR 0.75, 95% CI 0.71-0.79) than patients who were not eligible. Rural patients had 17% lower odds of MHV adoption compared with urban patients (OR 0.83, 95% CI 0.80-0.87) but substantially higher odds of CVT engagement and dual use (OR 2.45, 95% CI 1.95-3.09 for CVT and OR 2.11, 95% CI 1.81-2.47 for dual use).

#### Diagnoses Associated With My HealtheVet, Clinical Video Telehealth, and Dual Use

There were differences in MHV adoption, CVT engagement, and dual users across mental health diagnoses, holding all sociodemographic variables and comorbid mental health diagnoses constant. Patients diagnosed with major depression were more likely to be a MHV adopter than the veterans with other diagnoses. The odds of a veteran diagnosed with major depression adopting MHV were 25% higher than patients not diagnosed with major depressive disorders (OR 1.25, 95% CI 1.22-1.28). Patients diagnosed with PTSD had substantially higher odds of both CVT engagement (OR 1.74, 95% CI 1.58-1.91) and being a dual user (OR 1.86, 95% CI 1.77-1.96) than the odds for patients not diagnosed with PTSD. Finally, patients diagnosed with schizophrenia or schizoaffective disorder were significantly less likely than veterans with other mental health diagnoses to be a MHV adopter (OR 0.50, 95% CI 0.47-0.53) or dual user (OR 0.74, 95% CI 0.69-0.80).

## Discussion

### Principal Findings

This study is one of the first examinations of multiple (*dual*) health information technology use among veterans with mental health diagnoses. We explored an early period (2007-2012) of MHV use and CVT engagement among veterans with mental health diagnoses. Our findings suggest that during the study period, overall use or engagement with these health information technology tools was low and that dual use was exceedingly low. Among these veterans diagnosed with mental health conditions, differences in use by patient characteristics were like the differences previously reported in the overall veteran populations, with some exceptions [13]. To place this work into context, we have noted these exceptions below.

The unadjusted results suggest different patterns of early use of CVT and MHV across residential areas, gender, economic need, and race or ethnicity. Patients living in rural areas had higher odds of being CVT only or dual users, whereas those in urban areas were more likely to be MHV only users. Prior work has found higher use of MHV in urban veterans but has not explored dual use in rural veterans. This finding may be because of the emphasis on implementing CVT programs in rural areas and, thus, increased availability where they are more important for providing veterans with access to mental health services. It may also reflect differences in access to technology or the internet at home (used in accessing MHV) with greater availability in urban versus rural settings.

Although women represent less than 10% of veterans receiving VHA health care, the number of veterans who are women continues to grow. Female veterans had higher odds of being MHV adopters or dual users compared with male veterans. This finding is consistent with other literature showing that women VHA users are more likely to adopt patient portals than male VHA users [13]. Male veterans had higher odds of CVT engagement compared with female veterans. Although these findings describe a period of early use, recent research has highlighted the potential benefits of telemental health for women veterans [21] and the potential for it to reach this growing segment of VHA users [22] who have unique mental health needs. In a national CVT program for veterans with bipolar disorder, 19% of the participants were women [23]. Thus, our finding suggests there is an opportunity to increase the CVT engagement of women veterans through programs that address their specific needs.

Our findings suggest the presence of economic and race or ethnicity disparities in the use of eHealth technologies during the study period (2007-2012). Although the odds of CVT engagement were not associated with patient economic need, high economic need was inversely associated with MHV and dual use. African American and Latino race or ethnicity and high economic need were consistently associated with lower odds of use of MHV adoption or CVT engagement, even after adjusting for rural or urban residence and other demographic characteristics and diagnoses. The VHA experience is similar to that outside the VHA, as Roblin et al reported a gap in adoption of personal health records in the Kaiser Permanente Georgia patient population [24].

There were differences in MHV use and CVT engagement across mental health diagnoses. This study expands beyond prior work [13] that found veterans who had a diagnosis of major depression, PTSD, or bipolar disorder more often used any MHV feature and used MHV to refill prescriptions on the Web than veterans with other mental health diagnoses. Veterans with major depression or PTSD were high users of secure messaging as compared with other veterans with other mental health diagnoses. Patients who had diagnoses of schizophrenia or schizoaffective disorder were consistently less likely than patients with other mental health diagnoses to use any MHV feature, prescription refill, or secure messaging. They were also least likely to be dual users.

There is some evidence that CVT benefits veterans with serious mental illness. An analysis of a national CVT program for veterans with bipolar disorder showed positive effects across several domains, including patient engagement, clinical impact, and quality of care [23]. There is limited data available on the adoption and use of CVT in veterans with schizophrenia or schizoaffective disorder and the benefits of CVT on the outcomes of such patients. Kasckow et al conducted a review of telepsychiatry assessment and treatment in patients with schizophrenia (not just veterans) [25]. They reviewed internet, telephone, and video-based approaches. There were a limited number of studies in each modality, including a handful that explored video-based interventions. The CVT studies reviewed had limitations, and per the authors, the video-based modality showed initial promise with individuals diagnosed with schizophrenia.

### Future Research

It is important to uncover and address barriers patients face using eHealth technologies before new technologies are designed and implemented and before clinical interventions are delivered using those technologies. Future research could explore and then address differences in the barriers to use of patient-facing eHealth technologies, especially among veterans with mental health conditions. Such research should include exploring patterns of use of specific features or services across patients with different mental health diagnoses.

Understanding barriers and disparities could inform outreach efforts designed to increase adoption and use. For example, promotion and communication materials could be tailored to address specific barriers and disparities, and outreach efforts could include personalized and novel methods of training and education, including (1) members of a veteran’s clinical team teaching, reinforcing, and encouraging use of specific MHV features relevant to the veteran’s care such as accessing important illness, self-management, and provider visit content (eg, a medication list, a symptom checklist, or laboratory results from a recent visit); (2) creation of brief, step-by-step instructional videos of key MHV features and dissemination of these videos through the MHV portal and other means, including the VHA’s social media and public video-sharing websites; and (3) incorporating videoconferencing and/or screen sharing into customer service to help veterans learn and use MHV.

Future research should also explore more recent patterns of MHV use and CVT engagement, including dual use and complementary use among patients with mental health conditions. Since this study period (2007-2012), there have been major changes to MHV, including the full implementation of secure messaging and increases in patient adoption and use. In addition, the implementation of and engagement in CVT programs has grown. It would also be important to explore whether disparities in dual use and complementary use have changed. Future research should examine whether there are differences in mental health outcomes (eg, reduction in mental health symptoms, improvements in quality of life, and improvements in medication adherence) for different eHealth technology user groups.

Complementary use of eHealth technologies might support patient care, for example, prescribing providers (or their team) who use CVT might use secure messaging to remind patients about follow-up for important laboratory tests related to their treatment. As MHV allows users to track and self-enter a variety of information (eg, activity and food journals, vitals and readings, and allergies), providers and veterans who use manualized evidence-based treatments delivered using CVT might use MHV to support clinical treatment. MHV could host clinical program materials, and providers and veterans could share program materials using MHV secure messaging and could incorporate seamlessly MHV self-management tools (or program specific tools hosted on MHV) into clinical encounters.

### Limitations

There are several limitations to this study. Inherent differences in access to the health information technologies examined influenced our findings. MHV is a Web-based patient portal available to all VHA users, although actual access depends on factors such as computer or cell phone use and internet access. Unlike MHV, CVT is not available to all VHA users. It is behind the VHA firewall and not a publicly available Web-based tool. CVT programs are specific and vary by site; participation in them often requires travel (a known barrier to care) to a nearby VHA medical center or outpatient facility. Though there are CVT services conducted into the home, they comprise a smaller percentage of CVT telemental health encounters. Providers refer veterans to CVT programs, so veterans are not self-referred. As we did not have information about these veterans’ access to or familiarity with computers and cell phones, we could not assess their effects, if any, on MHV only or dual use. We were also unable to capture access to internet, which may be less in rural areas where the availability of broadband is much more variable. At the time of this study, information about MHV feature use was limited to the secure messaging and prescription refill features. Other features were not available or there was no tracking or capturing of information about their use. We also did not have information about the content or purpose of secure messaging such as the purpose of its use (ie, administrative or for mental health or medical condition management). Finally, the MHV secure messaging rollout to VHA mental health providers occurred in earnest after the study period; thus, the lack of mental health provider’s access to and use of MHV secure messaging before the rollout may have impacted adoption and use of MHV.

### Conclusion

Dual use has the potential to be highly beneficial for promoting access to care for patients with mental health diagnoses. Although CVT makes it possible to receive clinical care from remote clinicians, patient portal functionality such as secure messaging assists patients in communicating with their clinical providers between in-person or telehealth visits. Prescription refill functionality can support the clinical management of prescription medications and has the potential to improve medication adherence. Appointment viewing and scheduling functionalities can help patients schedule and obtain the in-person or CVT care they need. During this study, dual use was still exceeding rare. However, since 2012, both MHV use and the number of CVT encounters have increased. In fiscal year 2012, there were over 218,000 unique CVT encounters, and in fiscal year 2017, there were over 470,000 unique CVT encounters [14]. In addition, the VHA has introduced new technologies to support patient care, including VA Video Connect, a free new desktop computer, smartphone or tablet app. There is a nationwide implementation of this technology, which allows for telemental health encounters to take place from anywhere. VA Video Connect will exponentially increase the number of home telehealth visits in the coming years. The VHA is also implementing a secure SMS text messaging app for patient use in the self-management of numerous conditions. The goal is to enhance self-care using reminders, motivational texts, and educational protocols for numerous conditions (eg, weight loss and smoking cessation). Other mental health *apps* can be used independently or as part of therapy. Given the number of eHealth technologies, additional research is needed to understand how they can best be used in conjunction with one another to facilitate patient treatment. It will also be important to continue to monitor eHealth technology adoption and use by vulnerable patients to identify barriers to access and devise solutions to those barriers in the years to come.

## Abbreviations

CVT: Clinical Video Telehealth
eHealth: electronic health
HSR&D: Health Services Research and Development
MHV: My HealtheVet
OR: odds ratio
PTSD: posttraumatic stress disorder
QUERI: Quality Enhancement Research Initiative
TCT: telehealth clinical technician
VA: Veterans Affairs
VHA: US Department of Veterans Affair Health Administration
VISN: Veterans Integrated Service Networks
SMS: short message service
